# Intra-articular infiltration of adipose-derived stromal vascular fraction cells slows the clinical progression of moderate-severe knee osteoarthritis: hypothesis on the regulatory role of intra-articular adipose tissue

**DOI:** 10.1186/s13018-020-01664-z

**Published:** 2020-04-09

**Authors:** Juan Pedro Lapuente, Severiano Dos-Anjos, Alejandro Blázquez-Martínez

**Affiliations:** 1 Health Sciences PhD program, Universidad Católica de Murcia UCAM, 9 Campus de los Jerónimos n°135, 30107 Guadalupe, Murcia, Spain; 2 Lyposmol Biotech, Isabel Colbrand, 6, 28050 Madrid, Spain

**Keywords:** Knee osteoarthritis, Gonarthrosis, Adipose tissue, Mesenchymal stem cells (MSCs), SVF, Infrapatellar fat pad, Hoffa’s fat, Cartilage regeneration

## Abstract

**Background:**

The infiltration of the stromal vascular fraction (SVF) of autologous adipose tissue to treat osteoarthritis has been used for several years demonstrating its safety and noticeable efficacy. This article presents clinical data from patients afftected by moderate and severe knee osteoarthritis demonstrating safety and clinical efficacy of the treatment when this autologous cell product is injected in the knee joint and patients evaluated post-operatively after 1 year. However, what do we know about the mechanism that underlies this clinical improvement? This article proposes, for the first time in our opinion, a hypothesis of the mode of action that involves structural and molecular interactions between SVF and infrapatellar fat pad (IFP). As consequence, there would be a re-education of intra-articular adipose tissue, which we consider a key player for the clinical effect observed in the mid and long term mainly due to immuno-regulatory mechanisms.

**Methods:**

This is a retrospective and not controlled study that evaluated 50 patients (100 joints) ranging from 50 to 89 years old, separated by age cohorts. Clinical efficacy was assessed using the Lequesne, WOMAC, and VAS scales, by ultrasound control and quantification of the biochemical profiles of synovial fluid.

**Results:**

There were no serious adverse effects. All the indexes studied showed a significant clinical improvement after 1-year follow-up for all ages and OA degree groups. This finding was correlated with the ultrasound observations and biochemical data, which show a marked decrease in catabolic and pro-inflammatory molecules (MMP-2, IL-1B, IL-6, and IL-8) and significant increase for anabolic and anti-inflammatory molecules (IGF-1 and IL-10).

**Conclusions:**

We conclude that intra-articular SVF infiltration for knee OA treatment is safe and effective during 1 year. We propose that applied SVF cells cause a cascade of molecular and structural events that, through complex interactions between IFP and SVF, re-educating the intra-articular fatty tissue towards a homeostatic, protective, and anti-inflammatory function, which will ultimately promote the restructuring and regeneration of damaged tissues.

## Background

The EPISER study affirms that in 2020, knee osteoarthritis (OA) will be the fourth cause of disability in the world, and it is estimated that currently affects around 5 million people in Spain, being the chronic pathology that requires more health resources. It is estimated that the prevalence of gonarthrosis in Spain is 10.2% and that half of the population aged 50 years or older would have radiological signs of this pathology. For people aged between 60 and 69 years, the prevalence of symptomatic gonarthrosis is 28.6% and 33.7% in those over 70 years [1].

The different therapeutic options available to treat this pathology are complex, with more than 50 modalities of both pharmacological and non-pharmacological treatments [2], being the most common treatments the use of corticosteroid drugs, physiotherapy, injection of hyaluronic acid or platelet rich plasma. However, these traditional treatments, except the use of prostheses for very severe cases, have modest clinical efficacy and may have relevant adverse effects [3]. Moreover, these treatments fail to regenerate damaged articular cartilage but instead aim to reduce pain and/or maintain or improve joint function. Another option used with acceptable clinical results is autologous chondrocyte transplantation for chondral defects [4]. This option presents some disadvantages inherent to the chondrocyte’s own nature as well as the need for cell culture expansion: these cells are differentiated and produce fibrocartilage instead of hyaline cartilage [5]. On the other hand, the infiltration of both culture-expanded mesenchymal stem cells (MSCs) [6] and the stromal vascular fraction (SVF) of adipose tissue [7, 8] in osteoarticular pathology with degenerated cartilage has promising results. This new therapeutic approach using adipose-derived cells slows the disease progression and also improves joint function and pain without serious adverse effects, promoting a significant reduction of cartilage defects by regeneration of hyaline cartilage [6].

The immunomodulatory properties of adipose tissue MSCs are well known [9], but requires a homogeneous population of MSCs that meets the standards for their identification [10] and allows to determine exactly the number of grafted cells. Ensuring the reproducibility of the clinical procedure needs an in vitro cell culture expansion phase, and this is considered substantial manipulation, and thus classified as an ATMP (Advanced Therapy Medicinal Product), which implies the need to satisfy a rigorous regulation for its clinical use in Europe (EMA/CAT/852602/2018). To solve this limitation, there are medical devices commercially available on the market that allow the direct adipose tissue processing in the operating room to obtain SVF cells with minimal manipulation, so they are not considered as advanced therapies. The situation could be noticeably different in other countries. The regulatory framework stablished by the FDA in the USA is based on the 21 CFR (Code of Federal Regulations) 1271, and specifically the HCT/P (Human Cells, Tissues and Cellular and Tissue Based Products) 351. This process, in principle, requires an IND (Investigational New Drug) application that ends eventually in a Biologics License Application. However, different approaches are being discussed to facilitate these cellular treatments in North America: 21st Century Cures Act, accreditation standards for facilities or developing National Registries for Cell Therapies. Some Asian nations, such as Japan and South Korea, are optimizing their regulatory paths in some instances, to consider different evaluations based on adaptive licensing or conditional marketing approvals. Anyway, in all cases the interpretation of minimal manipulation and homologous use are the key concepts involved.

However, the amount of MSCs present in these cell preparations is usually lower than in treatments with culture expanded cells, although this does not necessarily imply less clinical efficacy [11]. Moreover, the heterogeneous cell composition of the SVF, which includes progenitors and differentiated cells of diverse origin (hematopoietic, endothelial, and stromal), might improve the treatment effectiveness. This phenomenon could occur through the maintenance of the present MSCs and their functions, as well as molecular and structural synergistic mechanisms. In fact, in studies with freshly obtained SVF, Traktuev et al. demonstrated that certain factors produced by the MSCs present in SVF, such as VEGF, help the migration and better survival of endothelial precursors (EPCs), which in turn produce PDGF-BB that allows MSCs to proliferate and migrate to the site of tissue damage [12, 13]. They also describe, both in vitro and in vivo, physical interactions between MSCs and endothelial cells (ECs) in which ECs form stabilized vascular structures due to the support of MSCs. Moreover, in 2011, Koh et al. demonstrated that direct subcutaneous SVF grafting can create a deep vascular network through disassembly and reassembly of blood endothelial cells at the implantation site, connected with the host vessels forming a functional circuit [14]. The SVF obtained from adipose tissue also contains a significant proportion of cells involved in immunoregulatory functions and vascular remodeling of hematopoietic origin. According to Morris et al. [15], macrophages (CD11b+) resident in rodent adipose tissue constitute 20% of the cells obtained in SVF, and of these, 70% are positive for CD301, a marker of M2 macrophages (with anti-inflammatory and proangiogenic function) [16]. Koh et al. described the role of SVF macrophages in vascular assembly, noting that macrophages were necessary for proper vascular-structural organization. There are other pathological and therapeutic scenarios that emphasize the roles of SVF immune cells. For example, in the fat grafting procedures performed by Dong et al. [17], the inclusion of SVF in the fat grafts leads to increased expression of CD206 (another M2 macrophage phenotype marker) and a negative regulation of the pro-inflammatory agents IL-1β and IL-6.

There are several studies that seek to identify the mechanisms of action of MSCs in OA, and many of them demonstrate the importance of the paracrine activity of these cells in the inflammatory process and the extracellular matrix remodeling, even suggesting that there is an activation of the anti-inflammatory effect of MSCs caused by the pathological, pro-inflammatory microenvironment of the lesion [18–21]. But in our opinion, there is no detailed model that describes the mode of action involving immunomodulatory mechanisms combined with structural and molecular synergies between the SVF and infrapatellar fat pad (IFP), the latter taken as the authentic paracrine regulatory body that is the key player in the development of the disease. From this new point of view, the present study aims to assess the clinical efficacy of SVF intra-articular injection in osteoarticular knee pathology, in 50 patients with bilateral knee OA (*n* = 100) divided by age cohorts, as well as discussing the possible underlying mechanism of action.

## Methods

### Study design and population distribution

This retrospective and not controlled clinical study describes the treatment of 50 patients (selected randomly) who attended our clinic between 2014 and 2016, who were administered SVF intra-articularly on both knees and completed the 1-year period of treatment follow-up (without control group). The patients presented grade III or IV knee osteoarthritis (bilaterally) according to the Kellgren-Lawrence scale, confirmed by clinical evaluation, ultrasound, and magnetic resonance imaging and/or radiography. All patients enrolled had failed with previous conventional treatments commonly used in the Spanish National Health System and had been recommended for joint replacement with a prosthesis. All the patients included in this study were informed in a personal interview by the medical staff with written information, after which they signed the consequent informed consents. All patients assumed the expenses related to the treatment, except those costs associated with the SVF processing device and the biochemical determinations in synovial fluid. Patients with severe local or systemic problems were excluded.

The group undergoing treatment was composed of patients aged between 50 and 89 years, affected by bilateral gonarthrosis grade III (50%) and grade IV (50%), of which 28 were men and 22 women, distributed by age cohorts (21 patients from 50 to 59 years, 17 from 60 to 69 years, 4 from 70 to 79 years, and 8 from 80 to 89 years) and physical assessment. The 50- to 59-year-old group contains 26 affected knees of grade III and 16 grade IV gonarthrosis, the 60- to 69-year-old group contains 18 affected knees of grade III and 16 grade IV gonarthrosis, the group of 70 at 79 years of age contains 4 knees affected by gonarthrosis grade III and 4 by grade IV, and the group from 80 to 89 years old contains 2 knees affected by gonarthrosis grade III and 14 by grade IV.

### Clinical outcomes

The results were evaluated using patient-reported questionnaires using the Visual Analogue Scale (VAS) assessment scale for pain and the Western Ontario and McMaster Universities Osteoarthritis Index (WOMAC) and Lequesne Index [22] for OA disease evaluation before treatment and at 3 months, 6 months, and 1 year after SVF infiltration.

The molecular profile of the synovial fluid was also quantified before treatment and at the end of the follow-up period (1 year), and subjective assessments were performed by anamnesis and clinical examination with the main objective of identifying possible adverse effects. On the other hand, the Spanish version of the CRES-4 scale [23] was used to assess patient satisfaction after treatment. An initial and final (after 1 year) articular ultrasound control was also included using an ultrasound device E-CUBE 7 (Alpinion Medical Systems Co., Gyeonggi-do, Korea), following the protocol defined by Grassi et al. [24]. Briefly, joint effusion was defined as the presence of hypoechoic content in the suprapatellar recess greater than 4 mm of anteroposterior maximum diameter. The effusion was subjectively quantified in 4 stages (0 absence, 1 mild effusion, 2 moderate, 3 intense). Likewise, 3 ultrasound parameters of the femoral articular cartilage were evaluated: clarity, integrity of the cartilage-soft tissue interface, and thickness in millimeter in the following 5 locations: external condyle, internal condyle, and immediately suprapatellar intercondylar recess and 1.5 cm above. The clarity of articular cartilage was classified semiquantitatively in 4 degrees: 0 = absence of internal echoes, 1 = minimal presence, 2 = moderate presence, and 3 = intense presence. The integrity of the cartilage-soft tissue interface was scored subjectively in 4 stages: 0 = normal, 1 = slight changes, 2 = moderate, and 3 = intense. The final score of the clarity, integrity, and thickness parameters resulted from the average of the scores in the 6 anatomical locations mentioned.

### SVF isolation

To obtain and process adipose tissue, the ADSC System commercial kit (Lyposmol Biotech, Madrid, Spain) was used in strict accordance with the manufacturer’s instructions. Briefly, each patient included in the study was performed a lipoaspirate under local anesthesia (tumescent Klein’s solution; all components from B. Braun, Melsungen, Germany) of the abdominal area in order to obtain 60 ml of adipose tissue to be subjected to enzymatic digestion with collagenase I and II in order to isolate, by centrifugation, the SVF. The resulting SVF was resuspended in Ringer Lactato (B. Braun), and the presence of active collagenase before infiltration was ruled out using the screening test included in the same kit.

### SVF characterization

As a quality control, SVF cells were counted using the trypan blue exclusion method and was used with the automatic counter TC20 (Bio-Rad, CA, USA) on a 10 μl aliquot obtained directly from the SVF sample.

For phenotypic characterization by flow cytometry, 0.5 ml aliquots were taken from three independent samples randomly selected to undergo erythrocyte lysis by hyposmotic shock (ZenBio, NC, USA) and then filtered through a 50-μm nylon mesh. The obtained cell solution was diluted in phosphate buffer without Ca2 + or Mg2 + (PBS, Merck KGaA, Darmstadt, Germany) 1:10 to obtain a diluted working cell solution, which was incubated for 15 min at room temperature and dark with the following fluorochrome conjugated antibodies: CD45-FITC, CD31-PE, CD34-PerCP, and CD146-PerCP (all from Sysmex, Kobe, Japan). For the exclusion of dead cells, DAPI was used, and for the exclusion of doublets the size and area parameters were faced. For the adjustments of the voltages and the compensations the appropriate controls were used (conjugated isotypes and markers one by one, respectively). For the acquisition of the samples, a BD FACSCelesta flow cytometer (Becton Dickinson, NJ, USA) equipped with the BD Diva software (Becton Dickinson) was used. Flowing software 2.5.1 (Perttu Terho, Turku, Finland) was used to analyze the results.

### Synovial fluid extraction and analysis of the molecular profile

As a complement to the clinical data observed during the follow-up of patients treated by intra-articular SVF infiltration, we quantified the synovial fluid levels of pro-inflammatory cytokines (IL1β, IL6, and IL8), anti-inflammatory cytokines (IL10), catabolic (MMP2), and anabolic (IGF1) factors, before and 12 months after the treatment. Briefly, 1 to 2 ml of synovial fluid was obtained using a 21G needle (Becton Dickinson). Immediately after extraction, it was frozen at – 80 °C in cryovials (Eppendorf, Hamburg, Germany) until quantification. ELISA tests were carried out following manufacturer’s instructions (all obtained from Wuhan Fine Biotech Co., Wuhan, Hubei, China) and quantified in a Microplate Absorbance Reader (iMark TM, BioRad, Hercules, CA, USA).

### SVF infiltration

The volume of SVF cell suspension per knee joint was adjusted to 7 ml using Lactated Ringer and then injected intra-articularly (and into the Hoffa’s fat or IFP) using ultrasound guided injection with a 21 gauge needle (Becton Dickinson).

### Statistical analysis

The significance (*p* < 0.05) of the data was analyzed with the Wilcoxon range non-parametric distribution test using Excel program (Microsoft, CA, USA). Each joint treated was taken into account independently for statistical analysis and clinical evolution.

## Results

### Clinical outcomes

In all scales used and age cohorts studied, we have found statistically significant differences between the baseline and the evaluation after 12 months (*p* < 0.05, non-parametric Wilcoxon test). Furthermore, the age or sex of the patients does not influence the clinical outcome. Thus, we decided to evaluate the global trend of the study group as a whole, regardless of age group or sex. As expected, the differences continued to be statistically significant for all scales.

**Fig. 1:**
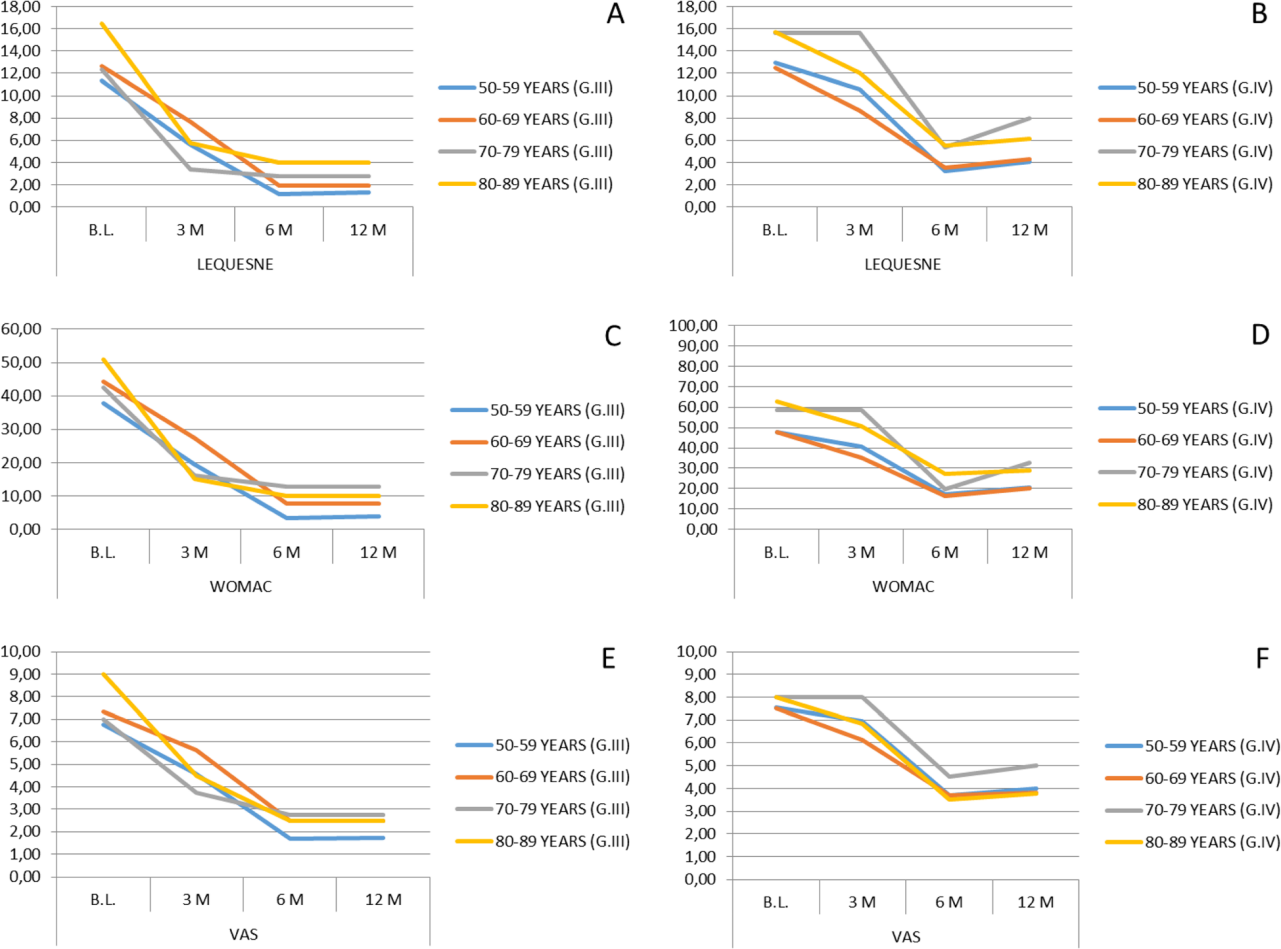
Clinical evolution of treated patients using Lequesne, WOMAC, and VAS scales after SVF treatment. Significant differences (*p* < 0.05, non-parametric Wilcoxon test) between the baseline and the evaluation after 12 months have been found in all age groups for all scales. Error bars are not included in plots due to high data variability and overlapping among them. B.L., Baseline; G.III/IV, OA grade III or IV; M, months

The initial mean values of the Lequesne index were 12.1 for OA grade III, and 13.77 for grade IV, registering final mean values of 1.76 at 12 months after implantation for grade III, and 5.05 for grade IV (Fig. 1a, b).

The total WOMAC score was 41.04 (mean values) for patients with grade III osteoarthritis and 52.8 for those with grade IV, obtaining a final average 12 months after SVF implantation of 6.18 for patients with OA grade III and 23.8 for grade IV (Fig. 1c, d).

The pain evaluation using the VAS index showed an important decrease in all age groups. The initial mean values were 7.08 for grade III osteoarthritis and 7.70 for grade IV, registering a final mean of said index of 2.12 at 12 months after implantation for grade III and 3.96 for grade IV (Fig. 1e, f).

If we analyze the evolution of pain by studying the average obtained in the scores of the three scales used in its assessment, we observe that in the group of gonarthrosis grade III there is a mean percentage decrease in pain of 74.44% at 12 months after performing the intra-articular SVF implantation. In the grade IV gonarthrosis group, an average percentage decrease of 54.11% of pain reduction was observed 12 months after the implant. The highest perception of improvement in the grade III OA group is maintained if we analyze the satisfaction index. The average patient satisfaction rate regardless of their age (expressed as a percentage) was 85.8% in the grade III gonarthrosis group and 76.2% in the grade IV gonarthrosis group.

**Fig. 2:**
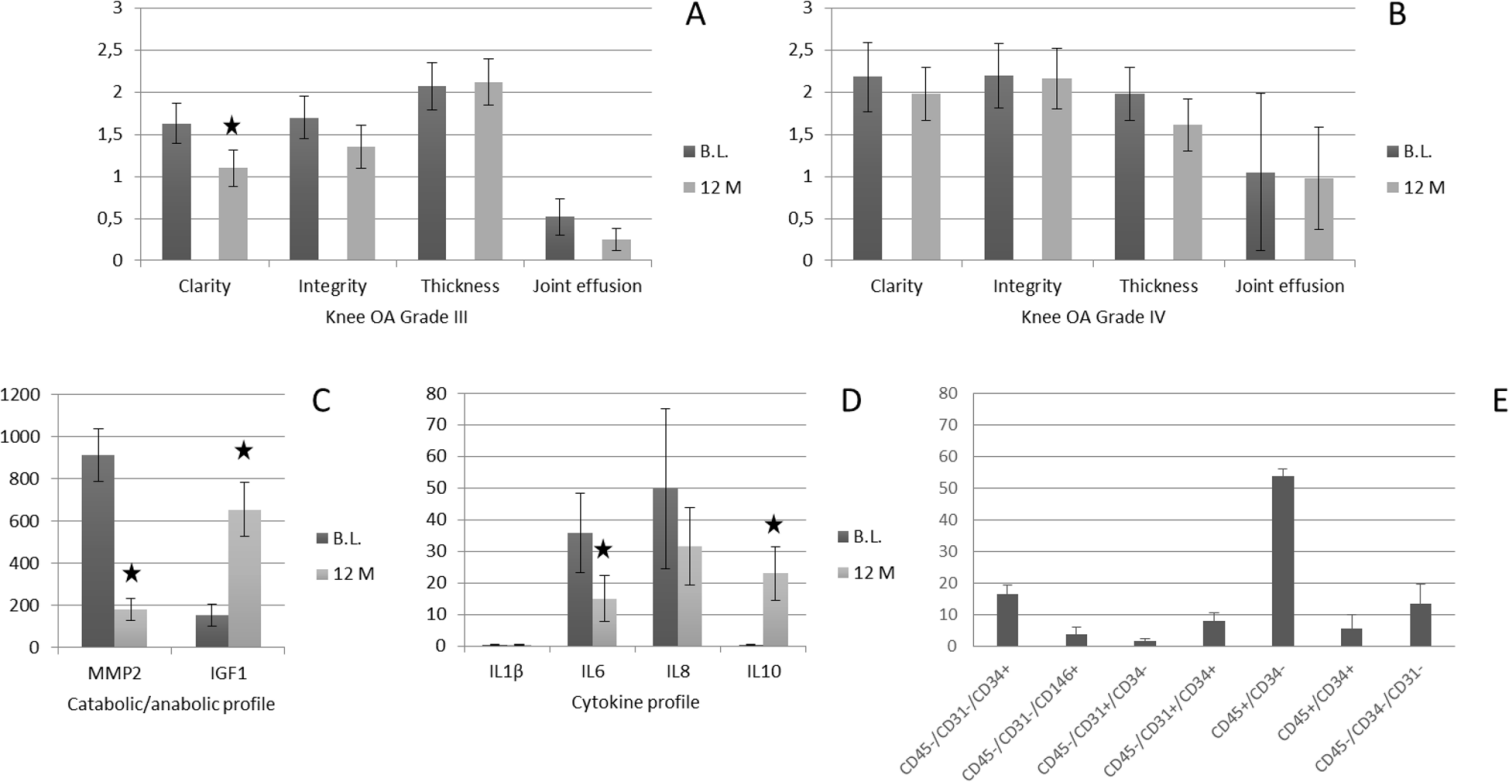
Evolution of diferent markers in knee OA treatment and SVF cell characterization. B.L., Baseline; M, months; black stars indicate significant differences (*p* < 0.05, non-parametric Wilcoxon test)

Regarding the ultrasound control, the study of the clarity and integrity of the soft tissue-cartilage interface showed a slight improvement in the grade IV group and a clear improvement in the grade III group. No significant changes were observed in any group with respect to thickness measurement. With respect to the ultrasound assessment of synovial effusion, an evident improvement was observed in the grade III group, being this improvement smaller in the grade IV group (Fig. 2a, b).

### Molecular profile of synovial fluid

The catabolic and anabolic profiles observed in synovial fluid by measuring the levels of metalloproteinase 2 (MMP2) and insulin-like growth factor type 1 (IGF1) decreased 80.24% and increased 330.64%, respectively. Likewise, analyzing the pro-inflammatory and anti-inflammatory profiles observed in synovial fluid by evaluation of pro-inflammatory cytokines (IL1β, IL6, and IL8) and anti-inflammatory cytokines (IL10), decreased by 32.26% in the case of IL1β, 58.25% in the case of IL6, and 36.77% in the case of IL8 and increased 70.80% in the case of IL10 (Fig. 2c, d). All results in the figures are expressed as picograms per milliliter of synovial fluid.

### SVF characterization

After doing a previous dilution, the number of nucleated cells present in the original samples was 3.21 × 10^6^ ± 0.44 × 10^6^ per milliliter, with an average vitality (viability) of 82 ± 9%.

The results of the phenotypic characterization of three representative SVF samples are reflected in Fig. 2e. The expression of different markers is showed in percent with respect to total nucleated cells.

## Discussion

### SVF clinical effect in knee OA

The use of SVF has great potential as a therapeutic agent in regenerative medicine, especially in human orthopedic applications. Preliminary clinical studies suggest that its use is safe and effective in the treatment of osteoarthritis [25, 26], which is reproduced in our study in which safety and efficacy have been evaluated by monitoring patients for 1 year. Adverse reactions observed were mild and transient, mostly abdominal discomfort related with liposuction procedure. As in other studies, we have not found severe complications in elderly patients [27]. Regarding the infiltration of SVF, the most frequent adverse effect was a mild and transient synovitis, probably caused by the volume injected, as described by other authors [28]. Some disadvantages of this technique are the processing time from tissue harvest to SVF isolation (60–90 min), and in some cases difficulties to obtain enough lipoaspirate volume from very lean patients (low body mass index).

Based on the type of clinical study carried, the inclusion of appropriate control group was not possible, and we understand that the data collected and possible conclusions drawn could be biased. Nevertheless, from a functional and pain point of view, the satisfaction rate of patients with grade III osteoarthritis was higher than that of patients with grade IV in all age groups, except in the older age group, which suggests that the more advanced degrees of the pathology entail a minor clinical improvement, or perhaps the expectations of the patients most affected by the pathology were higher. A more advanced disease stage would be also much more difficult to treat or respond to the treatment applied. In any case, in all age groups, all the indexes studied improved and remained so for a year, which demonstrates a clear reduction of pain and improved functionality in the knees treated with SVF. The results suggest that the most pronounced improvement occurs between the third and sixth months, but the assessments used are subjective, and we believe that drawing conclusions on this aspect could be premature. Other studies have reported an extended follow-up period of 2 years [29], finding similar positive results, although they suggest that efficacy may decrease after this initial period of treatment. There are still many questions to answer, such as the possibility of optimizing the therapeutic effect with repeated doses, the influence of cell dose used, or the possibility of testing different routes of administration, among others. Additional studies should also be carried out with or without platelet rich plasma [30], with or without hyaluronic acid [28], and even comparing SVF versus microfracture treatment [31].

### Synovial fluid evaluation and OA etiology

From a biochemical point of view, our results from synovial fluid analysis show a change in the molecular profile towards an anti-inflammatory phenotype and therefore are consistent with the observed clinical and ultrasound improvements observed. We were unable to find an equivalent study carried out under the same conditions to compare results, but other authors have also studied biochemical profiles in similar situations obtaining comparable results [32–37]. Moreover, even with a different treatment, for example, with hyaluronic acid, a change in the cytokine profile towards that typically anti-inflammatory phenotype is also observed [38]. All these results seem to indicate that the clinical improvement observed in OA, regardless of treatment, could be related with modified molecular profiles in the synovial fluid overtime.

### Infrapatellar fat pad as main driver of knee OA pathophysiology

Speaker and Fleshner suggested in 2012 that there could be a connection between the production of IL1β and the development of visceral fat [39]. It does not seem unreasonable to think that there could also be an answer to this mediator by the fatty tissue that constitutes the infrapatellar fat pad. On the other hand, Fain et al. in 2006 demonstrated that macrophages are primarily responsible for the secretion of pro-inflammatory mediators by adipose tissue [40]. In addition, other studies describe macrophages also as directly responsible for the inflammatory and destructive action in OA [41]. These macrophages can be found in synovial tissue, but also in the IFP, which makes them candidates for effectors of joint inflammation. In fact, the different concentration of adipokines in synovial tissue and blood plasma in OA [42] suggests that their production is local, that is, produced by intra-articular fatty tissue. Moreover, in 2011, Klein-Wieringa et al. demonstrated, by analyzing the Hoffa’s fat discarded when performing the prosthetic replacement, that it presented a pro-inflammatory phenotype and behaved like an authentic paracrine organ, generator of pro-inflammatory cytokines (IL6, IL8, and TNFα) that end up being related to the genesis and progression of knee OA [34]. In fact, IFP could have a leading role in the development of the disease, a hypothesis initially suggested by Ushiyama et al. in 2003 [43] and reconsidered years later by Distel et al. [44] and Clockaerts et al. [45]. Moreover, in the same study [34], they found differences in cell composition, such as in the macrophage immunophenotype and in the secretome, between the SVF of IFP and the SVF of adipose tissue subcutaneously, opening the door to relate the therapeutic effect in OA of SVF injection obtained subcutaneously with rebalancing the cellular and molecular composition of local adipose tissue. For all this, we deduce that the mechanism of therapeutic action of the intra-articular SVF implant could be mainly due to the immunomodulatory effect of the MSCs (an other immunocompetent cells present) of this cellular solution on IFP, which with improved homeostatic capacity, would regulate the progression of the disease and thus the clinical effect observed.

### SVF crosstalk

Native MSCs (in vivo) reside in the perivascular space in a quiescent state until chemical signals released from damaged tissues activate them, migrating to the site of injury and producing bioactive molecules that restore tissue homeostasis [46] by regulating the inflammatory response, reducing apoptosis, and recruiting progenitors of host tissue, in addition to direct differentiation into specific cells of the injured tissue. Therefore, theoretically, the infiltration of SVF into a pro-inflammatory environment would activate the MSCs (adaptive response) present in the SVF, which by producing mainly IL-1Ra (IL-1β antagonist), prostaglandin 2, IDO, IL-4, IL-10, and TGFβ, would modulate the immune cells present (local and grafted). This would produce a cascade of additive molecular interactions favoring immunotolerant and anti-inflammatory mechanisms. Polarization of macrophages to type M2, which in turn would express more IL-4, IL-10, and IGF-1, and would inhibit their production of IL-12 and TNFα. Inhibition of proliferation and activation of cytotoxic and effector T lymphocytes (inhibition of the production of TNFα and IFNγ) in favor of regulatory lymphocytes (Treg) related to immunosuppressive processes. Inhibition also of proliferation, differentiation, and migration (and therefore of the production of immunoglobulins) of B lymphocytes. Inhibition of the proliferation of NK cells (and therefore of IFNγ production) and reduction of apoptosis and ROS, by inhibition of neutrophils and preventing mast cell degranulation. Finally, they also seem to have an effect on dendritic cells as they increase their expression of IL-10 and reduce that of TNFα (reviewed by Brennen et al.) [47]. This entire chain of events has a direct effect on the reduction of metalloproteinase levels and results in the reduction of pro-inflammatory molecules and increase of anti-inflammatory ones in the joint environment (according to our analysis of the inflammatory profile of synovial fluid before and after treatment). This stops the vicious pro-degenerative circle, slowing the progression of the disease and restoring tissue homeostasis.

Although the MSCs seem to carry, in principle, the greatest biological potency, the therapeutic effect of SVF cells on the progression of OA with all cells present, and the synergies between them and the treated tissue can be also involved.

## Conclusions

We conclude that intra-articular SVF infiltration for knee OA treatment is safe and effective. We propose that applied SVF cells cause a cascade of molecular and structural events that, through complex interactions between IFP and SVF, re-educates the intra-articular fatty tissue towards a homeostatic, protective, and anti-inflammatory function. This mechanism would ultimately promote the restructuring and regeneration of damaged tissues.

It would be desirable to study and evaluate, in an animal model of knee OA and with labelled SVF cells, the progression at different times of histopathological changes in IFP, articular cartilage, and synovial membrane and fluid, to be able to understand in depth the observed therapeutic effect. This would allow to refute or confirm the proposed hypothesis.

## Data Availability

The data and materials are conveniently explained in the main text. If any reader needs any additional data, they can write freely to the corresponding author.

## Abbreviations

SVF: Stromal vascular fraction
IFP: Infrapatellar fat pad
MSCs: Mesenchymal stem cells
OA: Osteoarthritis
EPCs: Endothelial progenitor cells
PDGF: Platelet-derived growth factor
ATMP: Advanced Therapy Medicinal Product
VEGF: Vascular endothelial growth factor
ECs: Endothelial cells
IL: Interleukin
VAS: Visual Analogue Scale
WOMAC: Western Ontario and Mc Master Universities Osteoarthritis Index
MMP: Metalloproteinases
IGF: Insulin growth factor
TNFα: Tumor necrosis factor alpha
LIF: Leukemia inhibitory factor
DAMPS: Damage-associated molecular patterns
TLR: Toll-like receptors
IDO: Indoleamine dioxygenase
IFNγ: Interferon gamma
Treg: Regulatory T cells
NK: Natural killer cells
PTOA: Post-traumatic osteoarthritis
B.L.: Baseline
